# Cellular and Molecular Characterization of Microglia: A Unique Immune Cell Population

**DOI:** 10.3389/fimmu.2017.00198

**Published:** 2017-03-02

**Authors:** Carole Sousa, Knut Biber, Alessandro Michelucci

**Affiliations:** ^1^NORLUX Neuro-Oncology Laboratory, Department of Oncology, Luxembourg Institute of Health, Luxembourg, Luxembourg; ^2^Luxembourg Centre for Systems Biomedicine, University of Luxembourg, Esch-Belval, Luxembourg; ^3^Department of Psychiatry and Psychotherapy, Section Molecular Psychiatry, University of Freiburg, Freiburg, Germany; ^4^Department of Neuroscience, Section Medical Physiology, University Medical Center Groningen, University of Groningen, Groningen, Netherlands

**Keywords:** microglia history, Rio Hortega, technology, genome-wide, microgliome

## Abstract

Microglia are essential for the development and function of the adult brain. Microglia arise from erythro-myeloid precursors in the yolk sac and populate the brain rudiment early during development. Unlike monocytes that are constantly renewed from bone marrow hematopoietic stem cells throughout life, resident microglia in the healthy brain persist during adulthood *via* constant self-renewal. Their ontogeny, together with the absence of turnover from the periphery and the singular environment of the central nervous system, make microglia a unique cell population. Supporting this notion, recent genome-wide transcriptional studies revealed specific gene expression profiles clearly distinct from other brain and peripheral immune cells. Here, we highlight the breakthrough studies that, over the last decades, helped elucidate microglial cell identity, ontogeny, and function. We describe the main techniques that have been used for this task and outline the crucial milestones that have been achieved to reach our actual knowledge of microglia. Furthermore, we give an overview of the “microgliome” that is currently emerging thanks to the constant progress in the modern profiling techniques.

## Introduction

Until the beginning of the XXI century, the central nervous system (CNS) was seen as an immune-privileged site sealed by the blood–brain barrier, a barrier that was thought to prevent peripheral immune cells infiltration (1). Over the past years, thanks to rapid progress in concepts and new techniques, the idea of the brain as an immune-isolated organ has changed. Specifically, the recent discovery of a meningeal lymphatic system as a pathway allowing the trafficking of immune cells in the brain was the missing link between the brain and the immune system (2, 3).

**Figure 1:**
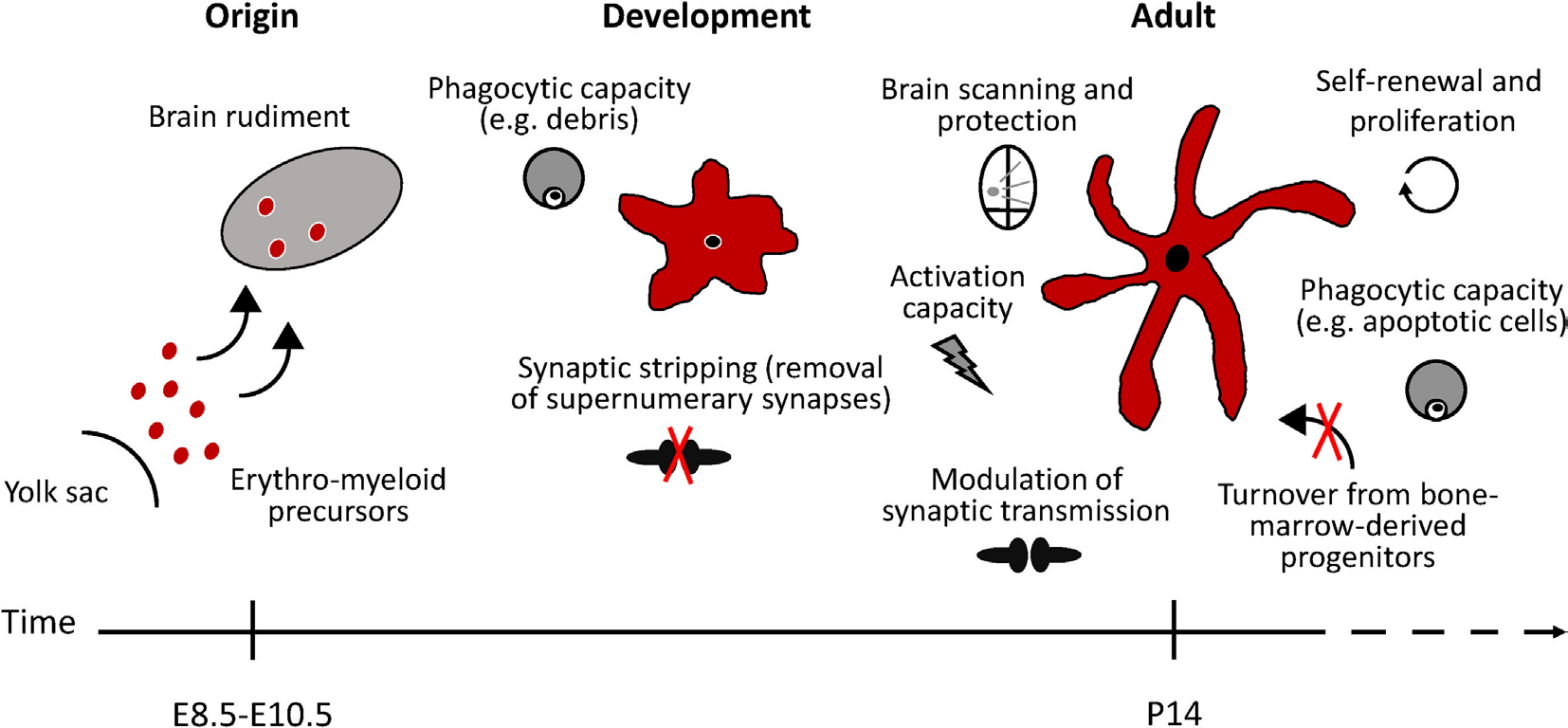
**Schematic representation of microglial functional states in the healthy murine brain**. Microglia arise from erythro-myeloid precursors in the embryonic yolk sac and populate the brain rudiment early during development. Microglial cell population is maintained by self-renewal, without the contribution of bone marrow-derived progenitors. In the adult healthy brain, microglia continuously survey the brain and readily react to any potential threat to the CNS homeostasis. Phagocytic microglia can detect and quickly remove damaged or dying neurons, preventing further damage to neighboring cells. During developmental stages, microglia phagocytic capacity is particularly important to prune supernumerary synapses. Microglia has also been suggested to modulate neuronal activity by influencing synapse transmission (synaptic stripping). Under specific conditions, microglia are able to remove dysfunctional synapses by physically interacting with functional neurons.

Distinct populations of resident macrophages colonize almost all the tissues in the body, including the CNS (4, 5). The CNS macrophage populations comprise microglia, perivascular macrophages, meningeal macrophages, and choroid plexus macrophages, though microglia are the only myeloid cells residing in the healthy CNS parenchyma (6, 7). Although sharing a common lineage with monocyte-derived macrophages, microglia’s unique ontogeny clearly distinguishes them from other myeloid cells. Microglia arise from erythro-myeloid precursors in the embryonic yolk sac and populate the embryonic brain early during development (embryonic day 9.5) (8). Unlike monocytes, which are constantly renewed from bone marrow hematopoietic stem cells throughout life, resident microglial cells in the healthy adult brain persist during adulthood *via* constant self-renewal without turnover from circulating blood progenitors (Figure 1) (8–10). Recent genomic and transcriptomic analysis additionally revealed the uniqueness of microglia, which possess specific genetic signatures that are clearly distinct from other brain and peripheral immune cells (11–20).

Microglia are non-neuronal cells belonging to the glial population of CNS cells. They comprise between 5 and 20% of the glial cells, approximately 10% of the cells in the brain, being as numerous as neurons (6, 21). Under physiological conditions, microglial cells play fundamental roles during neuronal development, adult neurogenesis, and in modulating synaptic transmission (Figure 1) (22–27). As the resident immune cells of the brain parenchyma, microglia act as central communicators between the nervous and the immune system, as they are the first sentinels protecting against invading pathogens and tissue damage (Figure 1) (28). The rise of innovative imaging, genetic, and immunological tools brought to light the remarkable high dynamism and plasticity of microglial cells under physiological conditions unmasking their crucial role in maintaining brain homeostasis. In the healthy mammalian brain, the so-called “resting” microglia are characterized by a ramified morphology, small cellular bodies, almost no cytoplasm, and slim branching processes bounded in fine protrusions. The majority of microglia occupies their own territory that does not overlap with the neighboring cells (29). Microglia are ubiquitously distributed throughout the adult CNS, yet they show regional diversities as they follow differences of high (such as substantia nigra) and low (such as cerebellum) densities (21). Two-photon imaging *in vivo* studies revealed that these ramified microglia are highly active, continuously extending and retracting their fine processes, and scanning the CNS microenvironment without disturbing the neuronal fine wire. This notable movement activity sets microglia as the fastest moving structures in the adult healthy brain, monitoring the entire brain parenchyma in less than four hours (30, 31).

Equipped with their branched morphology, microglia readily react to any potential threat to the CNS homeostasis, such as pathogens, trauma, or neuronal dysfunctions by undergoing morphological, genetic, and functional changes, usually referred as microglia “activation.” “Activated” microglia exhibit migratory, proliferative, and phagocytic properties as well as the capacity to release chemokines, cytokines, neurotrophic factors and to present antigens (28). Consequently, a proper and effective microglial function is crucial for CNS homeostasis not only under healthy conditions, but also during threatening events. Similarly to macrophages, in an attempt to simplify the intrinsic spectrum of microglial activation states, it has been assessed for several years that, under defined environmental stimuli, microglia adopt a “classical” (M1-like) or an “alternative” (M2-like) activation state, depending on the nature of the stimulus they encounter. As their corresponding states in macrophages, “classical” activated microglia have been associated with antimicrobial activity through a classical inflammatory reaction driven by the production of proinflammatory mediators, whereas “alternative” polarized microglia have been related to tissue repair and homeostasis restoration (7). However, such dichotomous paradigm represents the extremes of a large spectrum of activation states and is often related to inflammatory reactions and morphological changes, rather than reflecting the microglial physiological and functional status (32). Furthermore, at present, it is becoming more and more evident that “classical” or “alternative” activated microglia *per se* are barely present *in vivo* under healthy or diseased conditions (12, 33). In line with these evidences, concepts such as “resting” and “activated” microglia are currently considered simplistic and archaic as they do neither reflect microglia movement dynamism nor their functional plasticity [reviewed in Ref. (34–37)]. In this context, the microglia classification is currently reshaped in order to identify microglial cell phenotypes based, for example, on their inducing stimuli, such as M_LPS_ or M_IL4_ when stimulated, respectively, with LPS or IL4, instead of relying on microglia/macrophages pre-defined states (38, 39).

In order to follow this rapid advancement of microglia understanding and having in mind the magnitude to which accurate methodologies and innovative techniques can impact our knowledge, we aim here to review the breakthrough steps achieved in microglia research, from the past to the present days. This article is not meant to cover the history of microglia groundwork, but it is rather projected to highlight the steps that contributed to elucidate their identity, ontogeny, and function. We will specifically discuss the impact of the recent genome-wide expression profiling data, which revealed transcriptional microglial cells uniqueness when compared to peripheral immune cells.

## Finding and Characterizing Microglia

### The Concept of Neuroglia

**Figure 2:**
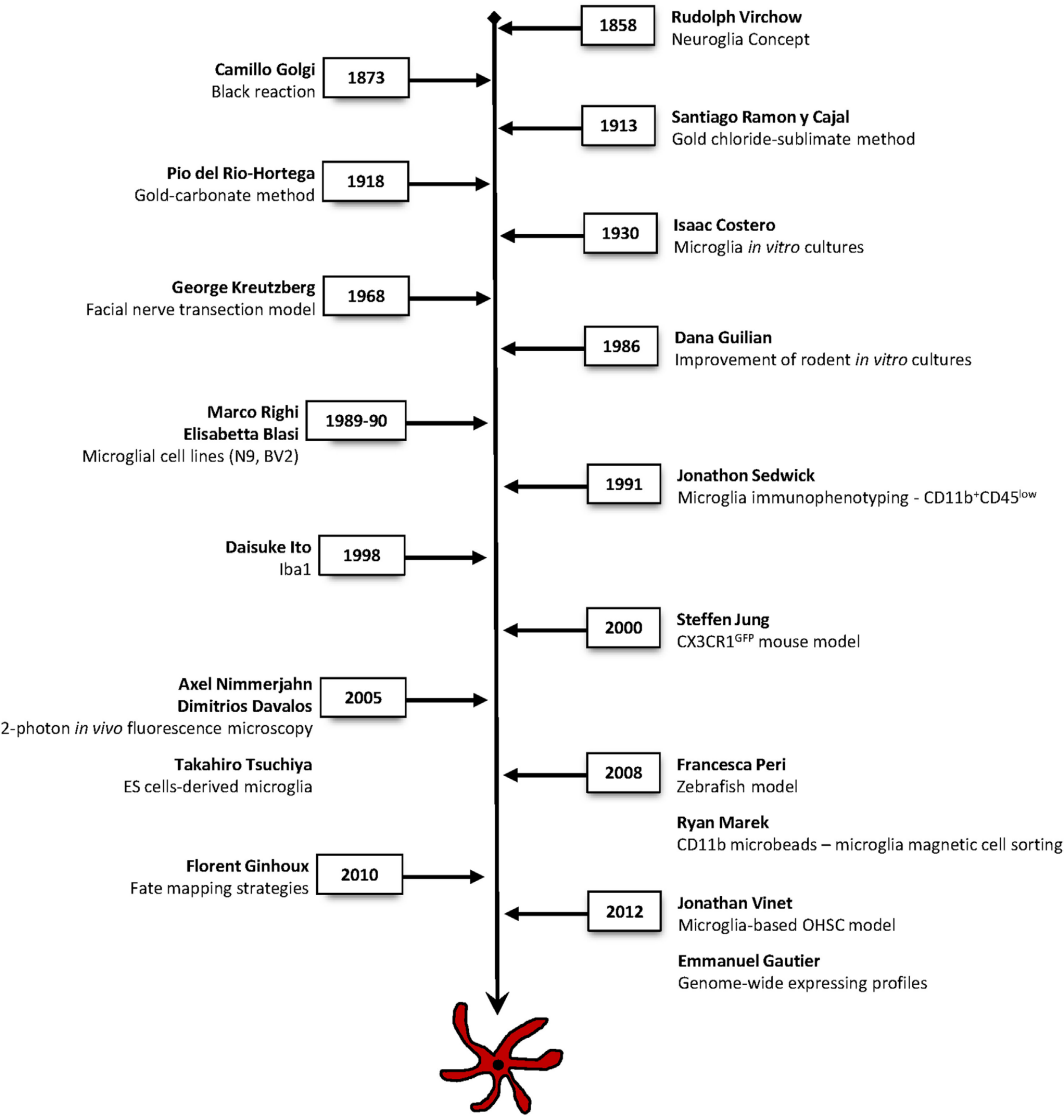
**Timeline of the main techniques and methodologies used in microglia research**. Major approaches that have contributed to breakthrough findings to elucidate microglial cells identity, ontogeny, and function.

How important is the historical context in shaping our current understanding of microglial biology? From the original challenge of “placing glial cells in the conceptual picture of the brain,” it is astonishing to realize that the same work conducted almost a century ago is still directing our present-day view, understanding and research of microglia (40). A timeline of the main tools and methods that revolutionized and critically contributed to elucidate microglial cells identity, ontogeny, and function is listed on Figure 2.

This journey has started in the mid-nineteenth century with the introduction of the concept of neuroglia (“Nervenkitt,” meaning nerve-glue) by Rudolph Virchow in 1856 [for historical review, see Ref. (40–43)]. Neuroglia consisted of a mesodermal connective tissue, the interstitial matter, likely of acellular origin, which major function was to fill in the spaces around the neurons and keep them together (“Zwischenmasse”). Such concept excluded any cellular elements within the neuronal tissue. Nevertheless, the first description of a neuroglial cell was a radial cell of the retina made by Heinrich Müller in 1851 (today known as the retinal Müller cell), years before Virchow introduced the concept of neuroglia (40, 41). Noteworthy, Virchow, Müller, and many others strikingly detailed neuroglia in their drawings using unstained tissue. Prominent discoveries regarding the morphological characterization and the cellular origin of glial cells arose from the inspiring efforts of histologists and anatomists such as Camillo Golgi (1843–1926), Santiago Ramón y Cajal (1852–1934), and Pio Del Rio Hortega (1882–1945).

### The “Third Element” within the CNS

In the early 1870s, a major breakthrough in neuroglia was empowered by Camillo Golgi and his famous “Reazione nera” (Black reaction) (44) [for more details, see Ref. (45)]. The paraffin sections and their microscopic observation allowed for the first time to see the whole neuronal and neuroglial tissue stained in black against a light yellow background. Although the staining did not permit to differentiate between neurons and neuroglia, it allowed to obtain the best images so far of neurons and neuroglia. Golgi was the first to represent the glial cells as a distinct cellular population from neurons (41). He also reported a diversity of glial cells in the white and gray matter and found that the glial processes contacted both blood vessels and nerve cells. The latter discovery led him to postulate the first theory regarding the glial function, that neuroglia was mostly responsible for the metabolic support and substance exchange of neurons. These functions were later on assigned to astrocytes (41, 45, 46).

Further progress in the study of neuroglia was made possible by Santiago Ramon y Cajal and his pupils. Although the name of Cajal is often associated with the “Neuron doctrine” of the brain structure using the Golgi staining to characterize neurons morphology of the neuronal circuits, Cajal laboratory arduously worked in deciphering the glial enigma by developing new staining and microscopic tools that allowed to identify, classify, and functionally characterize the glial cells. In 1913, Cajal introduced an improved technique, the gold chloride-sublimate staining, which significantly enhanced microscopic visualization under a light microscope of neuroglial cells specifically (47). Cajal’s method improved the visualization of the nucleus and other cellular elements, in contrast with the Golgi’s black reaction that did not distinguish these elements from the dark staining of the background. This method was selectively staining both protoplasmic and fibrous astrocytes. Using the gold chloride-sublimate staining and considering solely the morphological visualization, Cajal reported a new class of cells describing them as “corpuscles without processes,” thus introducing the controversial “third element” within the CNS to further discriminate this group of cells from neurons and astroglia, respectively the first and the second elements [for historical details, see Ref. (40–43, 45)].

Camillo Golgi and Santiago Ramon y Cajal were brilliant pathologists, and their merit and contributions to science were so notable that both were honored with the Nobel Prize in Physiology or Medicine in 1906 (48, 49).

### Microglia: “The True Third Element” Identified by Rio Hortega

The “third element” postulated by Cajal was later refined by one of his pupils, Pio Del Rio Hortega. As “what we observe is not nature itself, but nature exposed to our method of questioning” (Werner Heisenberg), a student of the Cajal school, Rio Hortega learned the staining methods of Golgi, Cajal, Achúcarro and others, and developed his own method, a modification of Achúcarro’s ammoniacal silver staining (43, 50). This approach allowed to selectively stain microglia and permitted to remarkably visualize glial cells under a light microscope (51). Using his famous silver-carbonate method, Rio Hortega has been able to deeply characterize the morphology of two independent cell types, which he called microglia and interstitial cells, later renamed by him as oligodendrocytes. This method allowed the visualization of the finer micelles that impregnated with prime detail the broad morphology of such cells (40, 41). Moreover, Rio Hortega realized that what Cajal reported as a new class of cells depicted as “corpuscles without processes” was in fact a limitation of his gold chloride-sublimate method that did not allow the complete observation of these cell processes (41). Henceforth, based on morphological and functional differences, Rio Hortega re-categorized Cajal’s “third element” as microglia, “the true third element,” placing oligodendrocytes together with astrocytes as the second element of the CNS. The level of details of his morphological observations as well as the sharpness of his drawings are amazingly resembling the ones that are obtained with the current strategies used to label microglia, such as immuno or transgenic labeling. Notably, Rio Hortega’s detailed cytological observations and statements are still rather valid today (28, 41, 52). Rio Hortega was one of the most important figures in microglia research; his visionary studies and technical brilliance have undoubtedly paved the foundations for the modern research of microglia.

In 1930, Isaac Costero, a prominent student of Rio Hortega, implemented the first *in vitro* culture method of microglial cells from human brain and recorded their activity using time-lapse cinemicroscopy [(53) for historical details, see Ref. (40, 42)]. His findings were particularly notable in that time since they already echoed microglia motile ability, a process that would only be clearly demonstrated almost one century later (30, 31).

### The Rise of the Modern Research

A modern era in microglia research occurred in the sixties, when George Kreutzberg in 1968 implemented the facial nerve lesion model in rodents to study axonal regeneration. The novel insights this model made possible included the study of microglial responses to injury without blood–brain barrier disruption, as well as the first observational differentiation of microglial responses from that of infiltrating mononuclear phagocytes. By using light and electron microscopic and auto-radiographic ultrastructure techniques, Kreutzberg aimed to identify the cells implicated in the regenerative process after the disruption of the facial nerve (54). Using this model, microglia and astrocytes were pointed as major players involved in peripheral nerve regeneration and degeneration. Thus, a major turning point came with the revelation that activated microglia physically interact with neurons by removing synaptic inputs. This feature, known as “synaptic stripping,” has been further described later in the cerebral cortex also by 3D electron microscopy (55, 56). These findings uncovered for the first time a potential neuroprotective role for microglia in neuronal regeneration. In the period preceding the introduction of this concept, microglial function was thought to be merely related to phagocytosis and to the engulfment of damaged neuronal cellular bodies (54, 57).

In the late 1980s, the immunohistochemical methods were replacing the conventional histochemical staining, which allowed not only to study microglial cell phenotypes and distribution, but also enabled the characterization of other cell types within the CNS (21, 58, 59). To specifically analyze microglial cells, it became urgent to be able to discriminate between microglia from brain monocyte-derived macrophages, since the main markers are shared between these cells. In 1991, Jonathon Sedgwick proposed an immunophenotypic discrimination through fluorescence-activated cell sorting (FACS) to distinguish parenchymal microglia (CD11b^+^CD45^low^) from other CNS macrophages (CD11b^+^CD45^high^) (60). The significance of this method was exceptional since it allowed for the first time to target microglia in their biological environment. Although not new, this approach quickly propagated the usage of discontinuous percoll gradients for isolating microglial cells from adult brains (60). Noteworthy, this technique is still the most used approach to specifically isolate resident microglia from CNS macrophages and infiltrating peripheral immune cells. In this context, a few years later, ionized calcium binding adaptor molecule 1 (Iba1) was reported to be expressed by mononuclear phagocytes and, within the CNS, by microglial cells. Iba1 was detected in all morphological and functional forms of microglia in both rodents and human (61, 62) and, since then, it has been widely used as a microglial marker *in vitro* and *in vivo*. Yet, recent expression profile studies revealed the expression of Iba1 in CNS macrophages, neutrophils, and monocytes (12), thus making the discrimination between recruited or infiltrating macrophages from CNS-resident microglia difficult.

### Tools to Study Microglia *In Vitro*

Although microglia cultures dated already from 1930 (53), it took 50 years to develop and improve *in vitro* methods to obtain and culture high numbers of microglia derived from rodent brains and to deeply study microglia biology (63), shortly after enriched cultures of astroglia and oligodendroglia being described (64). Henceforth, studies were developed to culture fetal and adult microglia (65–68). The development of *in vitro* cultures was indubitably an innovative tool that contributed to a greater characterization of microglia at several levels, which were not possible to be studied *in vivo*. This included the identification of polarization states, the interaction with other CNS cell types, the expression of neurotrophic and neurotoxic factors, pro- and anti-inflammatory cytokines, neurotransmitter receptors, and mitogen receptors (69–79). It was in this context that both microglia morphological transformation and immune functions were studied, and the concept of microglia as “pathological sensor” of the CNS environment emerged (80).

In the 1990s, protocols for isolating microglia from both neonatal and adult origin were developed. However, they were time-consuming and resulted in low yield of cells, which limited the functional study of microglia at the cellular and biochemical level. The most widely used method for the isolation of glial cells was the mechanical shake-off (64), which is based on differential adherence properties exhibited by the cells into the culture dish. Although several variations of this protocol were used worldwide, the introduction of a magnetic cell sorting using CD11b microbeads improved not only the yield of microglial cells in comparison to the shake-off method but also allowed the accurate separation of microglia from astrocytes (81, 82).

In an attempt to overcome the low yield and time-consuming approaches, new *in vitro* models, such as immortalized cell lines, started to emerge. In 1985, Blasi and colleagues successfully immortalized murine bone marrow-derived macrophages by infecting the cells with two retroviral oncogenes, *raf* and *myc* (83). Five years later, the same approach was adopted to develop the BV2 microglia cell line. The BV2 cells were shown to share several biochemical features of microglial cells (84). Chronologically, the N9 microglial cell line was the first to be generated *via* infecting primary microglial cells with the v-*myc* or v-*mil* oncogenes (85). Overall, the generation of microglial cell lines represented a new and limitless *in vitro* model for studying microglia properties. Since then, a variety of microglial cell lines from mouse, rat, or human have been developed and used for microglia research (Table 1). However, despite their universal usage, immortalized cell lines are prone to an increased inflammatory status and are sensitive to genetic drift and morphological changes. Additionally, recent studies have being pointing critical differences between these cell lines and primary microglial cells (86) as well as acutely isolated microglial cells (15, 87).

**Table 1:** **Overview of a variety of commonly used microglial cell lines**.

Name	Organism	Immortalization method	Reference
N9/N13	*Mus musculus*	*v-myc/v-mil* oncogenes	Righi et al. (85)
BV2	*M. musculus*	*v-raf/v-myc* oncogenes	Blasi et al. (84)
RBM129	*Rattus norvegicus*	SV40 large T antigen	Hosaka et al. (88)
CHME	*Homo sapiens*	SV40 large T antigen	Janabi et al. (89)
EOC	*M. musculus*	Spontaneous	Walker et al. (90)
C8-B4	*M. musculus*	Spontaneous	Alliot et al. (91)
MG5	*M. musculus*	p53-deficient mice	Ohsawa et al. (92)
MLS-9	*R. norvegicus*	–	Zhou et al. (93)
Ra2	*R. norvegicus*	Spontaneous	Sawada et al. (94)
HAPI	*R. norvegicus*	Spontaneous	Cheepsunthorn et al. (95)
HMO6	*H. sapiens*	*V-MYC* oncogene	Nagai et al. (96)
MG6	*M. musculus*	*c-myc* oncogene	Takenouchi et al. (97)
SIM-A9	*M. musculus*	Spontaneous	Nagamoto-Combs et al. (98)
IMG	*M. musculus*	*v-raf/v-myc* oncogenes	McCarthy et al. (99)

An alternative strategy to cell lines and primary cultures generated from neonatal or adult rodent brains took advantage of embryonic stem (ES) cells. Tsuchiya and colleagues described the first method to obtain microglial cells from ES cells *in vitro* Takahiro with Tsuchiya. Using a classical protocol to induce ES cells differentiation into tyrosine hydroxylase positive neurons, the authors succeeded in isolating a subpopulation of Iba1^+^ and CD45^+^ cells based on a density gradient (100, 101). The isolated population was shown to expand in high number and form mature microglial cells, to be functionally and morphologically identical to primary microglia, and to specifically migrate to the brain rather than to the periphery after transplantation. Of interest, this classical protocol of neuronal differentiation from ES cells displays neurogenesis (101) and yolk sac-like hematopoiesis (102), thus reflecting microglia development *in vivo*. Since these ES cells-derived microglia were not proven to proliferate or survive under culture systems, new improved protocols to explore the potential of these microglial precursor cells have been developed in the following years (38, 103–105).

### The Characterization of Microglia *In Vivo*

Culture-based systems have provided the majority of the knowledge about microglial biology. However, it is becoming unequivocal that, under these conditions, microglial cells loose much of their singularities, thus resembling more to a macrophage-like cell (15, 16). Therefore, elucidating the effective roles of microglia in the CNS requires the development of tools that allow their study and manipulation in their biological environment. Such approaches include, for example, genetic modification strategies. In the CNS, the expression of the fractalkine receptor (CX3CR1) is restricted to microglial cells, whereas the expression of its ligand, the fractalkine, is specific to neurons. Taking advantage of the specificity of CX3CR1, Jung and colleagues developed the first genetic animal model allowing to target microglia, the *Cx3cr1*^GFP^ knock-in mouse model (106). The murine *Cx3cr1* gene was replaced by a reporter gene encoding enhanced green fluorescent protein to generate a *Cx3cr1*-null locus. However, once more, gene-profiling data have recently shown that *Cx3cr1* is also expressed by other mononuclear phagocytes and that it is less microglia specific when compared with other recently identified microglia signature genes (15, 107). Moreover, since the insertion of the reporter gene in *Cx3cr1*^GFP^ mice generates a *Cx3cr1*-null locus, this approach resulted in mice heterozygotes or homozygotes for the fractalkine receptor (106). However, although homozygous CX3CR1 deficiency dysregulates microglial responses resulting in neurotoxicity (108), no microglial phenotype has been so far reported for heterozygote animals when compared to mice harboring the *GFP* transgene under the *Iba1* promoter (109, 110). Taken together, the use of this animal model is still having a tremendous impact, and opened the doors to the development of new genetic strategies to target microglia (111).

Another approach to circumvent the disadvantage of *in vitro* methods was the development of *in vivo*/*ex vivo* culture systems mimicking microglial behavior in their biological environment. Mouse organotypic hippocampal slice cultures (OHSC) were implemented in 1981 and, since then, they have been used and optimized as a model recapitulating an *in vivo*-like situation, thus allowing the study of the intrinsic functional and physiological mechanisms of the nervous system (112, 113). Taking advantage of the OHSC model, the laboratory of Knut Biber in Freiburg expanded their use and implemented this culture system to study adult microglia physiology (114, 115). In the OHSC, ramified microglia was recently reported to exert a neuroprotective function against N-methyl-d-aspartate-induced excitotoxicity (115). Most of the *in vitro* culture systems are based on the isolation of microglial cells from the brain, meaning that microglia is being studied outside of their biological context. In fact, much of the controversies relying on microglia behavior and function are likely the result of using experimental methods that are far from the physiological conditions. To overcome this obstacle, Masuch and colleagues improved their OHSC methodology by using microglial cells isolated from adult mice to replenish OHSC depleted of endogenous microglia. This model creates an *in vivo*-like environment that allows the functional study of different microglia phenotypes to be easily accessed *in vitro* (115, 116).

### The Discovery of Microglia Dynamics

For decades, microglia were seen as static cells displaying a “resting” phenotype under homeostatic conditions with the capacity to become “activated” when reacting to external stimuli (28, 35). As “the real voyage of discovery consists not only in seeking new landscapes, but in having new eyes” (Marcel Proust), this view has dramatically changed with the advance of microscopic tools that permit the “real-time” *in vivo* imaging of microglia in their physiological environment. Using a combination of the most recent tools at that time, such as the *Cx3cr1*^GFP^ mouse model and 2-photon *in vivo* fluorescence microscopy (a mildly non-invasive technique which achieves an imaging resolution of 100–200 μm of the mouse cerebral cortex), one of the most extraordinary findings that changed our knowledge about microglia biology was uncovered: microglial cells display a highly dynamic cell motility and plasticity in their fully ramified forms within the healthy brain, features that are unique to this cell type within the CNS (30, 31). Moreover, *in vivo* laser-induced CNS damage confirmed microglia morphological transformation and responses to local brain injury (30). It is worth to mention that, already in 1930, Costero suggested the motile nature of microglia, although the rudimental technology of his time limited definitive conclusions. It took 75 years since then to develop sophisticated microscopic imaging techniques to actually visualize microglia in their physiological environment, *in vivo*, and in three dimensions at the same time (31). Imaging studies were previously widely performed in brain slices to characterize microglial electrophysiological, morphological, biochemical, and pharmacological properties. Nevertheless, histological approaches did not allow to capture the interactions occurring within and between cells, the responses to stimuli, injury, or disease as well as the motile behavior of microglia (42).

Exploring the combined application of the 2-photon microscopy with microglia expressing fluorescent proteins *in vivo* will greatly continue to contribute to unveil crucial roles regarding the function of microglia (and other CNS cell types) in the healthy adult brain, but also in the developing and aged brain, and in several disease models (117).

As active surveyors of their environment, microglia serve critical functions in the homeostatic brain and have been shown to influence neuronal activity (30, 31). Therefore, dissecting the functional aspects of such processes requires a real-time observation of microglia performance in the living brain. In that regard, advances in imaging technologies associated with sophisticated transgenic cell labeling methods have greatly contributed to the understanding of microglial interactions with other cells. Because of their transparency, the zebrafish larvae and embryos offer a powerful *in vivo* high-resolution imaging of the dynamic interactions occurring within cellular and subcellular mechanisms (118). Interestingly, it also enables the visualization of the entire microglial cell network (25–30 cells) (119). In 2008, Francesca Peri, by generating lines of transgenic zebrafish that allowed differential labeling of various nervous system cells, developed a model for studying neuronal–microglial interactions. This approach demonstrated for the first time the dynamism of neuronal phagocytosis by microglia, in real time, and revealed the involvement of v0-ATPase proton pump in phagosomal-mediating vesicles fusion (120).

### The Origin of Microglia and CNS-Resident Macrophages

Microglial ectodermal vs mesodermal origin was a matter of extensive debate until recently. Upon Rio Hortega concept of “microglia” to distinguish oligodendrocytes from the true mesodermal elements, the ectodermal origin of glial cells was generally accepted (52). Given the phenotypical semblance of microglia to other macrophage populations, their myeloid origin was readily accepted. Even Rio Hortega himself believed that microglia could derive from blood circulating monocytes that invade the CNS, replacing the embryonic microglial cells. However, it was only recently demonstrated that microglia belong to the myeloid lineage (8, 10, 121). Using elegant transgenic fate mapping strategies to trace microglia precursors, it was revealed that microglia derive from primitive yolk sac myeloid progenitors that enter the CNS between embryonic days 8.5 and 9.5, rather than hematopoietic-derived cells. Briefly, mice expressing tamoxifen-inducible MER-Cre-MER recombinase gene under the control of the runt-related transcription factor 1 (*Runx1*) locus were crossed with a Cre-reporter mouse strain. A single injection of 4-hydroxytamoxifen given to pregnant females induces recombination in a 12-hour period, which leads to irreversible expression of fluorescent protein in RUNX1^+^ cells and their progeny in the knock-in embryos (122). Despite the fact that yolk sac, but also fetal liver progenitors express *Runx1*, at the embryonic day 7.5 only yolk sac progenitors are expressing *Runx1*, therefore, the tamoxifen-induced recombinase will only irreversibly tag yolk sac’s concomitant progeny. Moreover, the authors showed that adult microglia are maintained independently of definitive hematopoietic progenitors. To corroborate the notion that microglia are a distinct immune cell population independent from peripheral monocytes circulation, fate mapping strategies coupled with 12-color flow cytometry analysis were recently used to show that retinal microglia and monocyte-derived macrophages exhibit a distinct phenotypic signature. Moreover, this retinal microglia-specific profile (CD45^low^ CD11c^low^ F4/80^low^ I-A/I-E^−^) is stable under physiological or injury conditions (123). Such sophisticated methodologies are contributing greatly to decipher the unique features and functions of microglial cells under physiological and pathological conditions.

Similarly to microglia, it was recently shown that non-parenchymal CNS meningeal, perivascular, and choroid plexus macrophages are also derived from hematopoietic precursors, establishing stable populations throughout life span with self-renewal capacities (107). Among non-parenchymal CNS macrophages, meningeal and perivascular macrophages do not rely on circulating blood monocytes, while choroid plexus macrophages, which display a dual origin and a shorter turnover, partially depend on circulating blood cells (107). Yet, it is important to highlight that despite their ontogeny similarities, microglia are the only “immune” cells populating the CNS parenchyma, thus displaying unique features to serve critical functions associated to it. Nevertheless, non-parenchymal macrophages are strategically located at the brain boundaries and represent distinct and specialized populations of macrophages serving as key mediators for brain homeostasis and immune responses (107, 124, 125). Although it has been arduous to specifically target the different brain immune cells, perivascular macrophages have been recently discriminated from microglia by their expression of the mannose receptor (CD206) (107, 124, 125). Conversely, the P2Y12 receptor has been shown to be expressed by parenchymal microglia, yet absent in perivascular and meningeal macrophages (107, 126). Taking advantage of CD206 differential expression, it was recently demonstrated that both perivascular macrophages and microglia are the primary source of the chemokine CCL2, which mediates the infiltration of CCR2^+^ monocytes to the brain, exacerbating the neuroinflammatory environment occurring after status epilepticus (125). Moreover, it has been shown that perivascular macrophages mediate neurovascular and cognitive dysfunction induced by hypertension through their capacity to produce reactive oxygen species and inflammatory cytokines. Intriguingly, perivascular macrophages depletion with clodronate was sufficient to recover from neurovascular impairments (124). Specifically, Faraco and colleagues reported 60–65% reduction of CD206^+^CD45^hi^CD11b^+^ perivascular macrophages, yet the number of microglia and blood lymphocytes remained constant (124). As pharmacological strategies to efficiently deplete microglia are reported and used worldwide (127), such observations underline the need for discriminating between microglia and CNS-resident macrophages to understand their specific roles and clarify their truly homeostatic, neuroprotective, and neurotoxic roles.

### Genome-Wide Analysis

In the last decade, genome-wide sequencing technologies have become quite attractive for researchers and started to be widely used as powerful tools to decipher microglial unknown and unique physiological roles in either isolated or cultured microglia (11, 12, 14–20, 128–130). The Immunological Genome (ImmGen) Project was the first systematic study covering the expression profiles of murine macrophages from different organs. Using these data, Gautier and colleagues observed a high diversity among tissue-resident macrophage populations, suggesting their flexibility to adapt to their environment, but also revealed a unique expression profile intrinsic to microglial cells (11). This work opened the door for more sophisticated strategies allowing for the first time to identify a microglia-specific gene signature (15). This approach not only opens up the potential to understand how microglial cells behave in health and disease but also provides opportunities for the discovery and generation of genetic tools that could specifically target microglia. Moreover, applying such techniques to identify unique molecular signatures in both microglia and non-parenchymal macrophages are expected to challenge and shape our current understanding regarding the immune cell populations residing in the brain (107).

## A System Approach to Build the Microgliome

**Figure 3:**
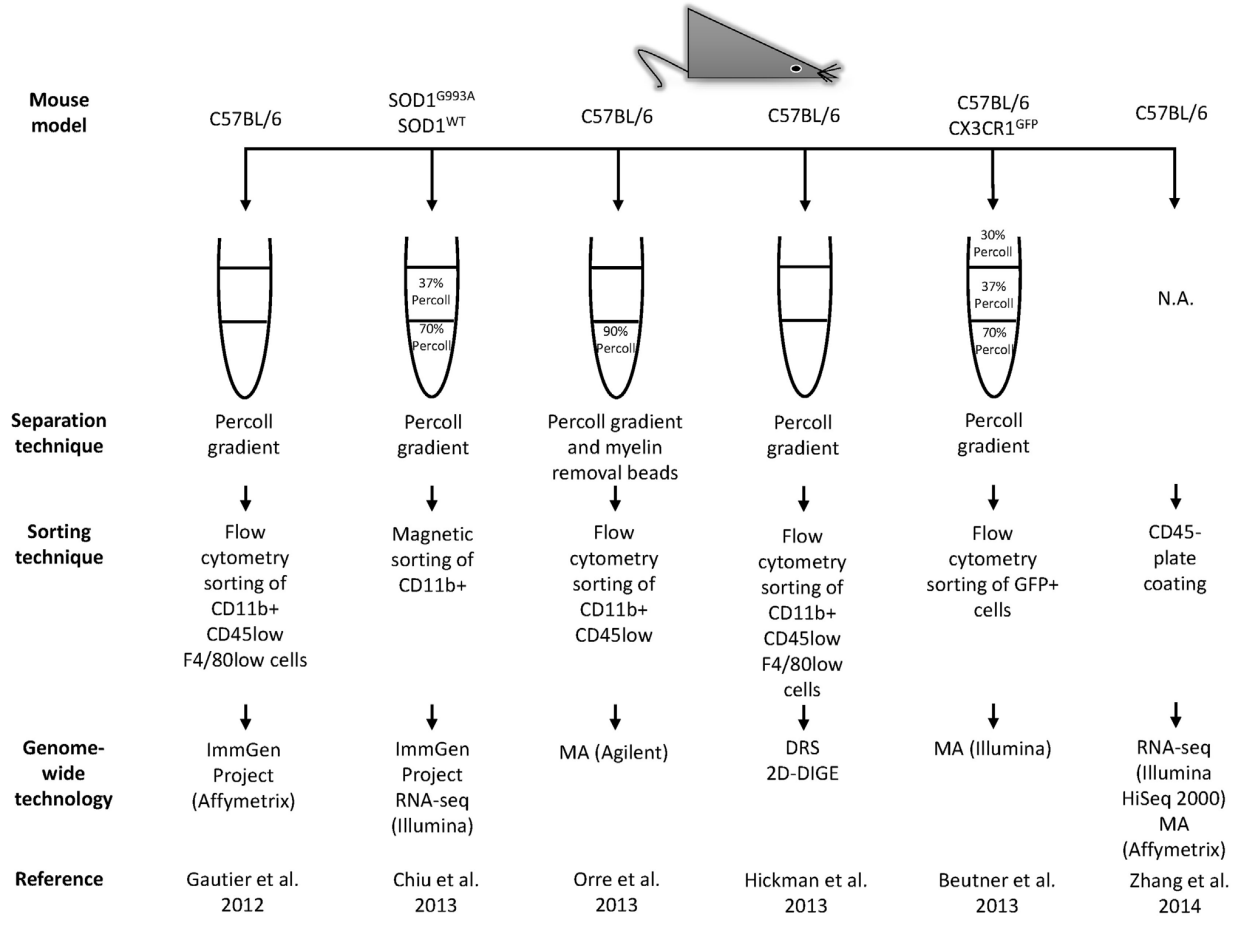

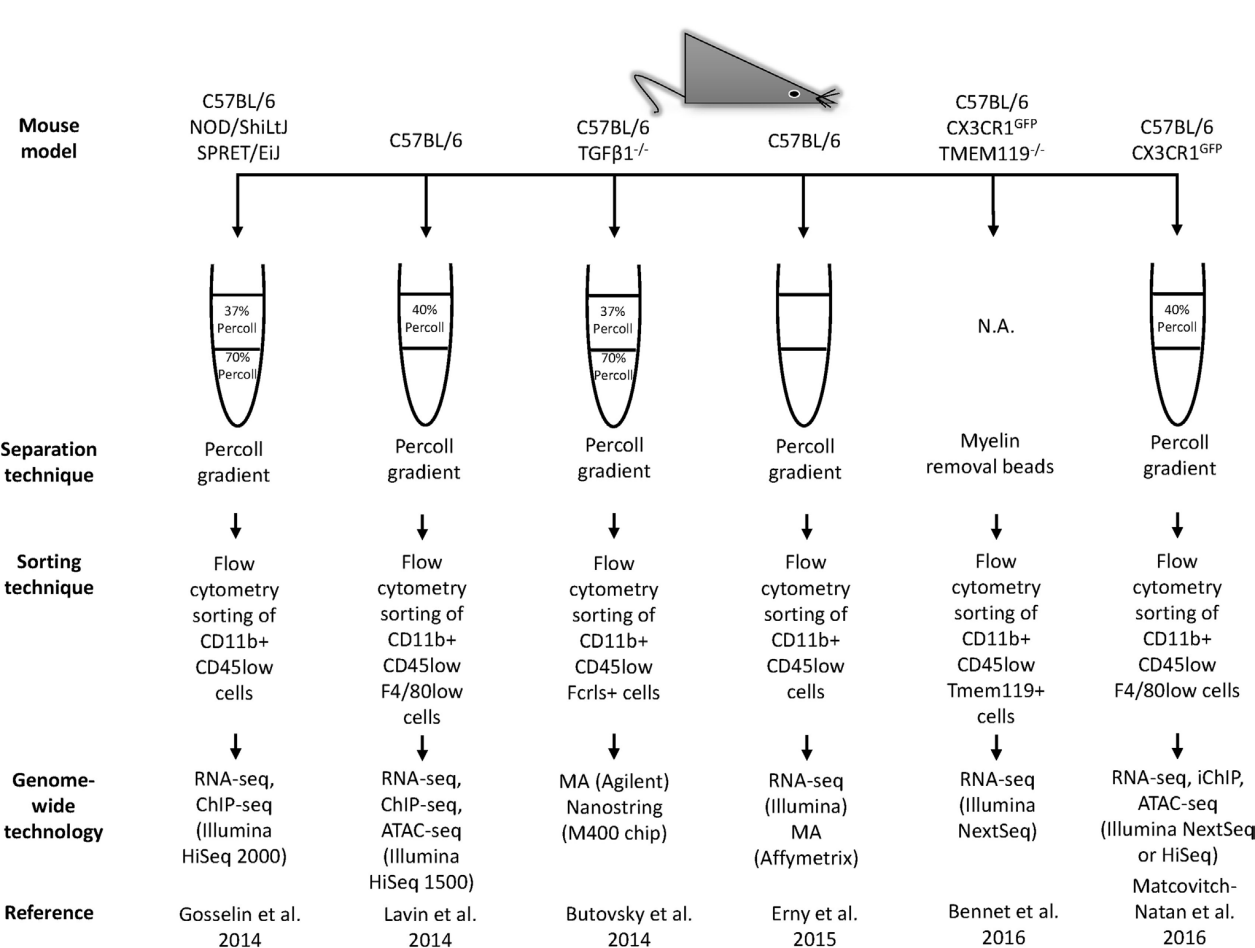
**Continued Workflow illustrating technical and methodological details used in the main genome-wide gene expression profiling studies**. The related results are shown in Table 2. MA, microarrays; RNA-seq, RNA-sequencing; DRS, direct RNA sequencing; 2D-DIGE, two-dimensional difference gel electrophoresis; ChIP-seq, chromatin immunoprecipitation sequencing; ATAC-seq, assay for transposase accessible chromatin; iChIP, indexing-first chromatin immunoprecipitation.

As the immune effector cells of the CNS, microglia are central in shaping homeostatic, neuroprotective, degenerative, and regenerative outcomes under different conditions. Recently, the use of modern sequencing technologies coupled with improved methods to enrich and to acutely purify microglia and other types of brain cells, greatly empowered the awareness of microglial uniqueness, properties, and complexity (Figure 3). These recent studies reveal a unique repertoire of transcripts selectively and specifically expressed by CNS-resident microglia that distinguish them from other CNS and peripheral cells, exposing other non-immune functions hitherto unforeseen of microglial cells (Table 2) (11, 12, 14–20, 128–130). The description of microglia gene signatures as well as their transcriptional changes associated with several brain diseases has also been recently accurately revised (131, 132). These systematic transcriptome datasets are fundamental to understand the gene expression patterns of both healthy and diseased tissues, thus allowing studies of brain cellular types aiding in elucidating the mechanisms associated to different CNS pathological processes, such as neurodegenerative diseases. For example, the microglia-unique gene signature is specifically modulated in normal brain aging compared to the young brain or under different neurological diseases (12, 14, 18).

**Table 2:** **Mouse microglia unique or highly expressed genetic profiles**.

Condition	Cell comparison	Omics	Microglia-selective or highly expressed genes	Remarks	Reference
Healthy	Brain mg vs p mØ red pulp mØ and lung mØ	Transcriptome Sensome	*Crybb1, Cx3cr1, Fcrls, Itgb5, Olfml3, Sall1, Siglech, Smad7, Socs3, Tmem119, Trem2*	Distinct molecular signatures among tissue macrophages Tissue-specific environment adaptation	Gautier et al. (11)

Disease (ALS)	Brain mg vs astroglia, spinal cord, NO, ph mØ and mo	Transcriptome Sensome	*Olfml3, Siglech, Tmem119*	Iba1 (Aif1) and CD68 not specifically expressed by mg *Cx3cr1* not as specific as *Olfml3, Tmem119, or Siglech* Mg genomic profiles likely to be disease-specific	Chiu et al. (12)

Aging	Brain mg vs astrocytes	Transcriptome	Young mg: *Ccl2, Ccl24, Ccl7, Ccr6, Clec4d, Ifitm1, Plbd1, Slpi* Aged mg: *Ccl3, Cd3e, Cd4, Lyz1, P2ry12, Pik3c, Rdh12Tnfs12, Tnfsf13b, Wbscr22*	Inflammatory responses associated with young mg 2.5 vs 15–18 months old	Orre et al. (18)

Aging	Brain mg vs whole brain, pmØ	Transcriptome Sensome Proteome	Mg sensome: *Ccr5, Cd53, Csfr1, Cx3cr1, Grp34, Hexb, Itgb5, Ly86, P2ry12, P2ry13, Selplg, Siglech, Tgfbr1, Tmem119, Trem2, Tyrobp* Mg transcriptome: *Adora3, Camp, Cxcr1, Fcrls, Gp34, Hexb, Olfml3, P2ry12, Rnase4, S100A8, S100A9, Siglech, Tmem119, Trem2* Young mg: *Adora3, P2ry12, P2ry13, Siglech, Tgfb1, Trem2, Tyrobp* Aged mg: Cxcr4, Ifitm, Il-1b, Lgals3, Tnf Mg proteome: *Ckb, Ldhb, S110-a9, Dpysl2, Eno3*	Mg phenotype shifts toward an alternative neuroprotective priming state (Stat3 and neuregulin-1) during aging 81% of the sensome genes are down-regulated during aging and involved in sensing endogenous ligands Normal aging distinct from neurodegenerative diseases 5- vs 24-month-old	Hickman et al. (14)

Healthy	ESdM vs primary mg, astrocytes, neurons, BMDC, BMDMs, T cells, brain mg	Surfaceome	*Cd40, Cmtm4, Krtcap2, Lpcat3, Lrp8, Mcoln3, Mfsd10, Pap2c, Slc30a5, Slco4a1, Stab1, Tmem48, Tmem55b*	ESdM and culture mg display similar transcriptomes	Beutner et al. (128)

Healthy	Brain mg vs neurons, astrocytes, oligodendrocytes, pericytes	Transcriptome	*Bcl2a1a, C1qa, Ccl2, Ccl3, Ccl4, Ccrl2, Cd83, Cebpa, Csf1r, Cst3, Cx3cr1, Gp34, Gp84, Hexb, Il1a, Irf5, Irf8, Itgam, Ly86, Olfml3, P2ry12, P2ry13, Plau, Rcsd1, Sfpi, Tgb1, Tmem119, Tnf, Trem2, Tyrobp* lncRNAs: *A430104N18Rik, Gm11974, Gm13476, Gm13889, Gm26532* Alternative splicing: *Ablim1, Actn4, Adam15, Arhgef1, Eif4h, Ktn1, Mcf2l, Mprip, Palm, Pkp4*	Glial cells display distinct genomic signatures Alternatively spliced RNAs are specific for each cell type	Zhang et al. (19)

Healthy	Brain mg vs large and small pmØ, TGEMs, BMDMs	Transcriptome Epigenome	*Cx3cr1, Grp56, Sall3* Mg-specific binding sites for PU.1: *Ctcf, Mef2, Nav2, Smad* (consistent with *Tgf-*β signaling) Mg-enhancer profile: *MaFb, Mef2, Smad3, Stat3, Usf1*	Freshly isolated mg- and large pmØ-specific gene expression profile greatly lost in culture Central nervous system environmental factors governs mg gene expression phenotype	Gosselin et al. (16)

Healthy	Brain mg vs kupffer mØ, spleen red pulp mØ, lung mØ, pmØ, small intestine mØ, large intestine mØ, mo	Transcriptome Epigenome	*Cx3cr1, Fcrls, Sall1, Siglech, Sparc* Mg-specific binding sites PU.1: *Mef2c* Mg-enhancer profile: *Mef2*	Myeloid cells display distinct chromatin landscapes Ontogeny and tissue microenvironment are critical to shape distinct mØ chromatin landscapes	Lavin et al. (17)

Healthy	Brain mg (E10.5–12.5; P4, P21, P30, P60) vs primary mg (P0-P1), cultured adult mg (M0, M1, M2a), human mg, blood monocytes, astrocytes, oligodendrocytes, hippocampal and cortical neurons, N9, BV2 and RAW264.7 cell lines, ESdM,	Transcriptome Surfaceome	*Csf1r, Cx3cr1, Fcrls, Gpr34, Gpr84, Hexb, Itgb5, Olfml3, P2ry12, P2ry13, PU.1, Sall1, Siglech, Socs3, Tgfbr1, Tmem119, miR-342-3p, -99a, -125b-5p* Surfaceome: *Fcrls, P2ry12*	*Tgf-β1* is central for adult microglia development *Fcrls* and *P2ry12* as prospective mg-specific markers (mouse/human) Mg-specific gene signature peaks between P21 and 2 months of age Freshly isolated adult mg-specific gene expression profile greatly lost in culture	Butovsky et al. (15)

Healthy	Brain mg	Microbiome	GF vs SPF mg: *Bcl, Ccnd3Cdk9, Csf1* (faintly increased), *Ddit4, Nfkbi-*α, *Sfpi1* (*Il-1*α, *B2m, Jak3, Stat1* decreased)	Host microbiota critically impacts microglia maturation, activation and homeostasis GF mg display a hyper-ramified morphology, increased density and increased proliferative rate Bacterial complexity critical for mg maintenance SPF vs GF vs ASF	Erny et al. (129)

Healthy (development)	Brain mg (E17, P7, P14, P21, P60) vs astrocytes, oligodendrocytes; newly formed oligodendrocytes, myelinating oligodendrocytes, endothelial cells, myeloid cells, BMDMs, human mg	Transcriptome	*Adora3, Cx3cr1, Fcrls, Gpr34, Gpr84, Hexb, Il1a, Ltc4s, Olfml3, P2ry12, P2ry13, Selplg, Tmem119*	*Tmem119* proposed as a stable marker for mouse (and human) mg *Tmem119* is limited to CD45^low^CD11b^+^ mg Mouse mg maturation occurs by P14 Tmem119 reactivity restricted to resident mg LPS-treated mg downregulates the expression of several mg-specific genes such *P2ry12, Tmem119, Fcrls, Olfml3, Ltc4s, and Adora3*	Bennett et al. (20)

Healthy (development)	Brain mg (YS; early mg: E10.5-E14; pre-mg: E14-P9; adult mg: from 4 weeks-old)	Transcriptome Epigenome Microbiome	YS: *Crc2Csf1, Ctsb, Dab2, F13a1, Lfit3, Mcm5* Early mg: *Arid3a, Psat1* Pre-mg enhancers: *Arid3a, Csf1, Psat1* Adult mg: *Crybb1, Egr1, Ets1, Fcrls, Fos, Irf8, Jun, MafB, Mef2a, Sall1*	Mg gene expression during development displays shifts in chromatin landscapes Microbiome impacts mg development (specifically adult mg), physiological function and likely influencing neurodevelopment disorders	Matcovitch-Natan et al. (130)

*Specific features and findings related to each study are specifically described. Genes are listed in alphabetical order. mg, microglia; mØ, macrophages, mo, monocytes; NO, neutrophils; DC, dendritic cells; p, peritoneal; ph, peripheral; BMDMs, bone marrow-derived macrophages; TGEMs, thioglycollate-elicited macrophages; ALS, amyotrophic lateral sclerosis; miR, micro RNA; lncRNA, long non-coding RNA; GF, germ free; SPF, specific-pathogen free; ASF, altered Schaedler flora; ESdM; embryonic stem cell-derived microglia; YS; yolk sac [adapted from Ref. (131)]*.

In the present context, to allow a fast and easy comparison of these genome-wide transcriptome profiles, new databases, such as Glia Open Access Database, available *via* www.goad.education have been established (133).

Driven by technological advancements, high-throughput -omics methods, such as genomics, transcriptomics, proteomics, and metabolomics seek the thorough description of changes in genes, transcripts, proteins, and metabolites, respectively. These system approaches have emerged as precious ways to interrogate several features of CNS uniqueness under healthy and diseased conditions. More importantly, they have made the molecular study of critical features of the different cell types that populate the brain possible.

Taking advantage of the data generated by these high-throughput studies, the “microgliome” is starting to emerge (Table 3). Here, we define the “microgliome” as the different -omics that characterize microglia under specific conditions, such as homeostasis, injury, or disease.

**Table 3:** **Overview of the emerging mouse “microgliome” profile**.

Epigenome	Transcriptome	Proteome
*Ctcf*	*Crybb1*	BIN1
*Irf8*	*Csfr1*	CKB
*MaFb*	*Cx3cr1*	LDHB
*Mef2*	*Fcrls*	LGMN
*Nav2*	*Grp34*	P2RY12
*Sall1*	*Grp84*	RGS10
*Sall3*	*Hexb*	S100-A9
*Smad3*	*Itgb5*	TPPP
*Stat3*	*Olfml3*	
*Usf1*	*P2ry12*	
	*P2ry13*	
	*Rnase4*	
	*Siglech*	
	*Slc2a5*	
	*Smad7*	
	*Socs3*	
	*Tgfb1*	
	*Tgfbr1*	
	*Tmem119*	
	*Trem2*	
	*Tyrobp*	
	*miR-342-3p, -99a, -125b-5p*	
**Reference**		
Gosselin et al. (16); Lavin et al. (17); Matcovitch-Natan et al. (130)	Gautier et al. (11); Beutner et al. (128); Chiu et al. (12); Hickman et al. (14); Butovsky et al. (15); Gosselin et al. (16); Lavin et al. (17); Orre et al. (18); Zhang et al. (19); Bennett et al. (20)	Hickman et al. (14); Butovsky et al. (15)

*Specific genes and proteins selected based on their high expression levels as described in the corresponding studies. Lists are in alphabetical order*.

At the epigenetic level, the gene expression profile of the environment-triggered signal-dependent specific factors are critical for microglia identity and are able to alter gene expression and gene enhancer profiles (16, 17, 130). Specifically, distinct tissue environments drive divergent programs of gene expression by differentially activating a common enhancer repertoire and by inducing the expression of differing transcription factors that collaborate with the macrophage lineage-determining factor PU.1 to establish tissue-specific enhancers (16). As previously mentioned, the microglia transcriptome is the unique set of transcripts expressed by microglial cells when compared to other brain and peripheral immune cells. The microglia proteome is defined as the unique array of proteins selectively expressed by microglial cells under homeostatic conditions (14, 15).

In some of the cited studies, the authors have started to list microglia-specific genes based on their cellular localization or function. In this regard, for example, Hickman and colleagues described a “microglial toolset for sensing changes in the brain’s milieu,” namely the “microglia sensome” (Table 4) (14). Similarly, the microglia “surfaceome” has been designated as the microglia-unique expression of cell surface molecules (15, 128). The “microglia sensome” is required to maintain the CNS homeostatic status and defines the threshold to which critical transitions between CNS homeostasis and pathology can be identified and compared. Highlighting the unique adaptation of microglia to the CNS parenchyma, the “macrophage sensome” is distinct from the one described for microglial cells (Table 4) (14).

**Table 4:** **Microglia and macrophage sensome**.

Microglia-specific sensome genes	Macrophage-specific sensome genes
*Camp*	*Ahnak*
*Crybb1*	*Alox15*
*Cx3cr1*	*C4b*
*Fos*	*Cd5l*
*Fcrls*	*Cfp*
*Grp34*	*Crip1*
*Grp56*	*Cxcl13*
*Hexb*	*Ecm1*
*Hpgd*	*Ednrb*
*Itgb5*	*Emilin2*
*CMRF35-like molecule*	*F5*
*Ngp*	*Fabp4*
*Olfml3*	*Fcna*
*P2ry12*	*Fn1*
*P2ry13*	*Gm11428*
*Rnase4*	*Icam2*
*S100a8*	*Msr1*
*S100a9*	*Pf4*
*Siglech*	*Prg4*
*Slc2a5*	*Ptgis*
*Slco2b1*	*Retnla*
*Syngr1*	*Saa3*
*Tgfbr1*	*Serpinb2*
*Tmem119*	*Slpi*
*Trem2*	*Thbs1*

*Top 25 sensome genes described by Hickman and colleagues (14) exclusively expressed in microglia (left column) or in macrophages (right column). Genes are listed in alphabetical order*.

Furthermore, emerging data have been highlighting a brain-gut crosstalk that is critically modulated by the gut microbiota (134). The microbiota consists in the specific microflora that colonizes an established microenvironment, such as the gut. Recently, the impact of the host gut microbiota on microglia homeostasis has started to be addressed. Such studies support a critical role for host microbiota in shaping microglia maturation and immune function. The microglia-specific microbiome is defined as the host bacteria that maintain and shape microglia maturation, development, and functions, encompassing microorganism-induced transcriptomic changes in microglial cells (129, 130). These studies address the impact of host microbiota in microglia under specific conditions, such as germ-free and specific-pathogen-free environments. Specific genes that are precisely affected by the host microbiota conditions have been identified (*Bcl, Ccnd3, Cdk9, Csf1, Ddit4, Nfkbi-*α, *Sfpi1*) (129, 130).

As a perspective, the “microgliome” will be built based on the data generated at different -omics level in order to associate specific microglial phenotypes to each defined condition, such as homeostasis, injury, or disease. Moreover, the fact that microglia exist under several forms, are highly dynamic and highly sensitive to specific cues, emphasize the importance of compare whether their profile is closely reflected when they are cultivated *in vitro*. Thus, *in vivo* vs *in vitro* “microgliomes” are also foreseen.

## Concluding Remarks

Since the description of Rio Hortega’s staining method to distinguish microglia from the surrounding cells, advanced techniques and tools over years and decades have contributed to elucidate the origin of microglial cells as well as their functions during development and in the adult brain under healthy or diseased conditions. Recently, molecular profiling of freshly isolated adult microglial cells have once and for all shown that microglia are distinct from other immune cells of the CNS as well as from other mononuclear phagocytes. The unique features of these cells are essential to fulfill their critical functions in their environment. The distinction of microglia from other myeloid cells is fundamental for understanding their specificity in brain development, function, and disease. Difficulties in addressing microglia functions were mostly related to limitations in isolation protocols and lack of techniques or specific markers to specifically target these cells. Genome-wide profiling of acutely isolated cell populations have opened up new opportunities for the identification of microglia-specific markers distinct from other myeloid cells to precisely address microglia functions (20). For these *ex vivo* techniques, the significance of isolating microglia from their context (135) as well as the fact that microglia processes, which possess fundamental functions, might be lost during their isolation procedure (131) must be still taken into account.

It is believed that many CNS diseases are strengthened by inappropriate microglial cell functions, therefore understanding the specific molecular triggers and analyzing the resultant gene expression signatures that characterize microglial cell phenotypes is a fundamental step. Supporting this hypothesis, the generated genome-wide profiles suggest that microglia are likely to display unique and characteristic gene signatures depending on the CNS disease [for revision, see Ref. (131, 132).]. In this context, transcriptomic profiles of mouse microglia from distinct brain regions revealed a considerable regional immune-phenotypic diversity across the adult lifespan and an interregional dependency on microglial aging phenotype (136). Microglial expression profiles were also shown to be subtly distinct between different rat brain regions (137). Yet, microglia maintain a specific core signature that, independently of the brain regions, differentiates them from other macrophages (136). This unique microglia transcriptional profile has been recently described in zebrafish (138) and in human microglial cells (20, 126, 139). Taken together, these genome-wide studies are deeply contributing to uncovering the involvement of microglia in different CNS processes and are opening the doors to the identification of microglial specific targets that may have potential therapeutic values (140).

In summary, it has been shown *in vivo* that microglial activity is critical for normal brain development (27). *Ex vivo* microglial gene expression profiles from different physiological or pathological conditions show that they scarcely resemble to those classified as the classical polarization states (12, 33), while *in vitro* microglia studies highlight their immune properties (7). Overall, the notion that microglia share a myeloid origin with other macrophage populations, thus inheriting features such as phagocytosis abilities, lead to the questioning about the real meaning of experimental observations on microglia as the “immune effectors” and “professional phagocytes” residing in the adult CNS. Are microglia immune features a result of their myeloid origin or an outcome of *in vitro* observations? Are microglial cells essential for CNS host defense or are they professional phagocytes fundamental for brain development? Which cells are the real immune cells of the brain? Further investigations, taking advantage of the technological progress, need to be carried out to properly address these open questions. For example, single-cell transcriptomic and proteomic technologies are gaining their momentum to directly access gene and protein expression profiles (141–143). Their application to individual microglial cells will certainly uncover new magnitudes of cell heterogeneity and will contribute to further characterize microglial phenotypes and functions under physiological and disease contexts.

## Author Contributions

CS and AM conceived and wrote the manuscript. CS prepared the tables. CS and AM created the pictures. CS, KB, and AM critically revised and approved the final version of the manuscript.

## Conflict of Interest Statement

The authors declare that the research was conducted in the absence of any commercial or financial relationships that could be construed as a potential conflict of interest.
